# LIMPRINT: Prevalence of Chronic Edema in Health Services in Copenhagen, Denmark

**DOI:** 10.1089/lrb.2019.0019

**Published:** 2019-04-22

**Authors:** Susan Nøerregaard, Susan Bermark, Tonny Karlsmark, Peter J. Franks, Susie Murray, Christine J. Moffatt

**Affiliations:** ^1^ Copenhagen Wound Healing and Lymphoedema Centre, Bispebjerg University Hospital, Copenhagen, Denmark.; ^2^ Centre for Research and Implementation of Clinical Practice, London, United Kingdom.; ^3^ School of Social Sciences, Nottingham Trent University, Nottingham, United Kingdom.

**Keywords:** chronic oedema, lymphedema, primary Lymphoedema, secondary Lymphoedema, prevalence, LIMPRINT, Denmark

## Abstract

***Background:*** The International Lymphedema Framework developed an international study, Lymphedema Impact and Prevalence International (LIMPRINT), to estimate the prevalence and impact of chronic edema (CO) in heterogeneous populations.

***Methods and Results:*** A validation study using the LIMPRINT methodology was undertaken in Denmark. Participants with CO were identified from inpatient services and compared with those identified within a specialist lymphedema service and three primary care settings. Of 452 inpatients available for screening, CO was present in 177 (39%) and absent in 275 (61%). In addition, 723 participants were found from specialist and primary care services (LPCSs). Inpatients were significantly older and more likely to be underweight or normal weight. They were more likely to suffer from heart failure/ischaemic heart disease (44.6% vs. 23.4%, *p* < 0.001) and have neurological problems (18.1% vs. 10.9% *p* = 0.009). Patients in the inpatient group were nearly all suffering from secondary lymphedema and were less likely to have a cancer or venous diagnosis, but more likely to have immobility as the cause of CO (44.0% vs. 17.7%, *p* < 0.001). No inpatients had midline CO compared with 30 within LPCSs. Fewer in the inpatient group had standard CO treatment (17.1% vs. 73.5%, *p* < 0.001) and subjective control of swelling was worse (19.9% vs. 66.7%, *p* < 0.001). While the inpatient group experienced fewer acute infections, when they did so, they were more likely to be admitted to hospital for this (78.6% vs. 51.0%, *p* = 0.049).

***Conclusion:*** The prevalence of CO in inpatient facilities is high and those with CO have multiple comorbidities that vary according to setting. The feasibility study showed that the methodology could be adapted for use in different health systems.

## Introduction

Chronic Edema (CO) is a common health problem that has received scant attention in health care systems despite its impact on the patient. Internationally, the terminology, CO, has now been adopted to replace the term lymphedema, which implies that it is only related to a lymphatic problem. CO has been developed as a public health term to capture the wide range of patients affected, which includes patients with primary lymphedema as well as those with a range of secondary forms. The term CO indicates chronic swelling for at least 3 months, irrespective of the underlying etiology.^1^

The correlation between CO and cancer is well documented within prevalence and incidence studies. A meta-analysis concluded that 20% of women surviving breast cancer will develop breast cancer-related CO despite recent improvements in treatment such as sentinel node biopsy.^2^ The perception that CO is purely related to cancer has now changed, and it is recognized that secondary forms of CO may affect many patients and this study aims to examine this in more detail. In the original study using the definition of CO, the authors found that the overall prevalence was 1.3 per 1000 rising to 10.3 per 1000 in the elderly in the health care population, indicating a strong association with age.^1^ More recently, a study using the same methods indicated that the prevalence had risen to nearly 4/1000.^3^ It is known that there is a strong correlation between obesity and CO, although robust studies on this are required to establish the true size of the problem and understand the mechanisms causing CO.^4^

It has been shown that CO has a significant impact on the individual, families, and the health care system.^5^ CO most frequently affects limbs and is associated with pain, reduced mobility, and complications such as cellulitis and wounds, which may require hospitalization. Untreated, the condition can lead to elephantiasis where the limb increases drastically in size with significant tissue changes. It is predicted that CO will increase in the coming years because of higher average life expectancy, higher cancer survival rates, and increase in levels of obesity.^6^ Changes in demographics in both Western and Asian populations indicate that the human and economic costs of CO and its complications will be a much larger health problem in the future. As in other countries, knowledge about the size of the problem in Denmark, the types of patients affected, and the impact it has on patients and the health care system are unknown.

This study formed part of an international epidemiology study, Lymphedema Impact and Prevalence International (LIMPRINT), to define the prevalence and impact of CO in health services in different countries and health care systems. This study was the initial validation project undertaken following development of the study methodology.

To understand the current scale of the problem within health services in Copenhagen, Denmark, and to explore the feasibility of using the LIMPRINT methodology, the study was designed with three related aims:

- to determine the prevalence of CO within a range of health care settings in Copenhagen, including two hospital inpatient services, patients treated in a specialist lymphedema clinic (acute care based), and three primary care settings,
- to compare profiles of patients identified in different settings, and
- to explore the feasibility of undertaking the study to inform the wider implementation of LIMPRINT during the international roll-out phase.

The study used the following patient inclusion and exclusion criteria:

Inclusion

- Patients over 18 years of age who had CO for more than 3 months
- Able to understand written information in Danish
- Able to give informed consent to participate in the study

Exclusion

- Patients who did not want to participate
- People under the age of 18 years
- People who did not understand Danish
- Edema present for less than 3 months

### Data sets

The development and validation of the LIMPRINT methodology have been published separately.^7^ During the study, the following data were captured using standardized questionnaires:

- social demographics and care: age, gender, and main care provider
- presence and site of CO using a body map, details, and duration of CO, cellulitis history, leg ulceration and wounds, pitting test, Stemmer's sign, and soft/hard tissues
- classification of the CO into primary/secondary lymphedema and related risk factors
- lower limb mobility status (bedbound, chairbound, walks with walking aid, or walks unaided)
- upper body mobility (full range of movement, limited range of movement, or normal function)
- obesity status (underweight, normal weight, obese, or morbidly obese)
- relevant comorbidities: diabetes mellitus, neurological disorders, heart failure/ischemic heart disease, or peripheral arterial occlusive disease
- types of treatments received for CO
- subjective control of swelling, access to specialist service, and requirements for community care
- details of swelling

As the study documentation was written in English, the first step involved ensuring an accurate translation into Danish. This involved translation and back translation of all study documentation. The data collection tools were then piloted with 10 patients at the wound healing center by general nurses to ensure that all questions were easily understood. Documentation was also completed by specialist nurses on the same patients to identify any discrepancies in the data captured between the groups.

## Ethical Issues

The study was approved by the Danish ethics committee j.nr: 2007-58-0015. Confidentiality and anonymity were guaranteed to participants before entering the study. All patients were invited to participate in the study and received verbal and written information. Patients unable to provide consent were excluded. Participants could withdraw from the study at any time.

## Study Setting

The study was divided in two parts.

- A point prevalence study carried out among inpatients at two acute hospitals in Copenhagen, over a period of 4 days.
- Patient identification from four different sites in the Copenhagen area through professionals was undertaken over a 4-week period. These services were an acute-based lymphedema service, primary care nurses in Copenhagen, primary care nurses in a rural setting, and a private therapy-led lymphedema service in primary care.

### Hospital inpatient prevalence study

In addition to ethical approval, permission was granted by the hospital administration to conduct the study. The head nurse of each ward was informed in writing about the study and they received a plan for the time of the visit from the research team. A list of the number of beds available at each ward in addition to bed occupancy and a list of current patients were available. Reasons for patients not being available at the time of the visit were recorded and reasons for any other exclusions.

In addition to the main inclusion and exclusion criteria for the study, the team asked the head nurse to identify any patient who should not be approached and the reasons for this. Patients were provided with written information 4 hours before being approached for consent. Participants were informed that they could withdraw from the study at any time.

### Screening for CO

All patients were screened to identify the presence or absence of CO by the use of a standard assessment, the pitting edema test, which measures the site and depth of swelling in an edematous area when pressure is applied by the thumb. A positive result is indicated if a pit remains following removal of pressure. This is a well-established clinical technique, but inter-rater reliability was further assessed for its use in the LIMPRINT study. Levels of agreement were found to be high between general nurses performing the technique and a clinical expert (gold standard) in Japan.^8^ Edema was judged to be chronic if it had been present for 3 months or more.

## Research Team Preparation

The research team consisted of student nurses who were supervised by specialist teams from the wound healing center at Copenhagen and visiting international lymphedema consultants. The specialists were responsible for classification of patients into a primary or secondary form of lymphedema in all cases identified with CO. Any participant for whom the underlying classification of lymphedema was uncertain was discussed within the expert group and a second clinical assessment was undertaken.

During the study period, small research teams were formed with experts in each group. All those involved in data collection were educated and were assessed to be competent by specialists working with them during the first assessment of participants. Student nurses did not classify patients as cases. All those involved in data collection received a day of education and were assessed using a competency framework.

The inpatients were examined over a 4-day period and data entered into a paper-based case report form. Due to time and limited resources, a random permuted block design allowed for a 50% minimum sample in which additional clinical, health-related quality-of-life, and disability data were collected.

Quality mechanisms and study monitors were in place to ensure that data were complete before leaving the clinical area and to avoid double counting of any participants who may have moved wards during the study and may have been screened twice. At the end of each day, data for the day were checked by an independent quality monitor. After data capture was complete, they were subsequently entered into the online electronic data system, which has additional quality procedures to ensure complete and accurate data.^7^

### Primary care and specialist lymphedema services

Staff in each primary community nursing team screened their total caseloads for consent and inclusion, irrespective of the reason for needing nursing care. The study was carried out by nurses with expert knowledge in wounds and CO. Training was provided for all sites using the procedures outlined above. A unique patient identification code was issued for each patient to avoid double counting, and all questionnaires were preprinted with participant ID numbers. Master identifier lists were retained by each service manager to ensure that anonymity was maintained and for data protection purposes. Two specialist nurses were responsible for assessing the quality of data and entering into the international database.

### Data management and statistical analyses

All data collected were coded before being keyed into a database designed for the study. The datasheet was stored securely and destroyed after statistical analyses had been completed. Information from the database was downloaded to an Excel file. This file was then uploaded into a standard statistical package (Stata 12.0; Statcorp). All analyses were undertaken using univariate comparisons between the groups. Continuous data were analyzed using a *t*-test for comparison. Categorical data were compared using the Pearson *χ*^2^ test. Where numbers were small in one or more cells, Fisher's exact test was used.

## Assessment of Feasibility

At completion of each day during data collection, the study team met together to discuss any issues they had faced and the solutions they used when necessary. This included assessment of methods used to screen patients in busy ward settings, difficulties of using the randomization process, and the practical challenges faced by research teams. A field diary of these issues was kept by the principal investigator who shared the overall findings with the international research team who were able to make additional tools available for participants in the wider research study.

## Results

### Hospital inpatient prevalence

Within the potential hospital patient population of 578 patients, 452 were included for screening for CO. From the total group, 126/578 patients were excluded. The main reason was being absent from the ward at the time of screening (*n* = 76) or an inability to consent due to issues such as dementia (*n* = 50). Of the total participants (*n* = 452) who were screened, 177 (39%) suffered with CO for more than 3 months and 275 (61%) did not.

### Lymphedema service and primary care nursing services

A total of 723 participants with CO were identified through other services. The specialist lymphedema service identified 410 (57%), primary care nurses in Copenhagen identified 253 (35%), primary care nurses in a rural setting identified 51 (7%), and 9 (1%) were from a private lymphedema clinic in the community.

### Comparison of age and gender

Table 1 gives details of the age and gender of participants in all groups. While age was similar between men and women in the lymphedema service and primary care nursing services (LSPCs), there was evidence of a substantial difference between genders in the different services. The age of both genders was significantly higher in the inpatient group for both women (<0.001) and men (0.006). There was a slightly higher proportion of women (55%) in the LSPC group compared with inpatients (51%), but this did not achieve a standard level of statistical significance (*p* = 0.32).

**Table 1.** Demographic Details and Comorbidities of Participants with Chronic Edema

	*LPCS*			*Hospital inpatient prevalence*			t *(df)*	p
	N	*Mean*	*Sd*	N	*Mean*	*Sd*		
Age (women)	390	66.6	18.4	90	76.5	11.8	4.90 (486)	<0.001
Age (men)	325	66.4	15.3	87	71.3	12.6	2.75 (410)	0.006
Total	715			177				

	N	*%*	N	*%*	χ^2^*(df)*	p
Gender						
Female	398	55.1	90	50.9	1.01 (1)	0.32
Male	325	44.9	87	49.1		
Total	723		177			
Obesity						
Underweight	26	3.6	21	12.0	25.00 (3)	<0.001
Normal weight	446	61.7	89	50.9		
Obese	192	26.6	56	32.0		
Morbidly obese	59	8.2	9	5.1		
Total	723		175			
Diabetes						
Present	144	19.9	43	24.3	1.65 (1)	0.198
Absent	579	80.1	134	75.7		
Total	723		177			
Heart failure/IHD						
Present	169	23.4	79	44.6	32.18 (1)	<0.001
Absent	554	76.6	98	55.4		
Total	723		177			
Neurological disease						
Present	79	10.9	32	18.1	6.73 (1)	0.009
Absent	644	89.1	145	81.9		
Total	723		177			
Peripheral arterial disease						
Present	61	8.4	21	11.9	2.01 (1)	0.16
Absent	662	91.6	156	88.1		
Total	723		177			
≥ one comorbidity	456	63.1	141	79.7	17.52 (1)	<0.001
No comorbidity	267	36.9	36	20.3		
Total	723		177			

IHD, ischaemic heart disease; LPCSs, lymphedema and primary care services.

### Level of obesity

As would be expected, there was a proportion of participants who were either obese or morbidly obese. In the inpatient group and LSPC group, percentages of patients considered to be obese or morbidly obese were, respectively, 37% and 35%. Being underweight occurred significantly more in the inpatient group (12%) compared with only 4% in the other services. Overall, there was evidence of a significant difference in distribution of BMI categories (*p* < 0.001), Table 1.

### Other comorbidities

There was a high percentage of participants in both groups with comorbidities associated with CO, Table 1. Heart failure/ischaemic heart disease was significantly more prevalent in the inpatient group (45% vs. 23%, *p* < 0.001) as well as neurological disease (18% vs. 11% *p* = 0.009). Similar nonsignificant differences were noted for the presence of diabetes and peripheral artierial occlusive disease. In these numbers, more than one comorbidity can occur in the individual patient. There were significantly more patients suffering from at least one comorbidity (including obesity) in the inpatient group compared with the LSPC group (80% vs. 63%, *p* < 0.001).

### Classification of lymphedema

Lymphedema was diagnosed and classified as either primary or secondary from the patient history of CO, Table 2. There was a significantly higher percentage of patients with primary lymphedema in the LSPC group (13% vs. 1%). Of the 175 patients with secondary disease in the inpatient study, only 1 (0.6%) had cancer-related lymphedema compared with 55 (9%) in the LSPC group (*p* < 0.001). Of the total number of patients with secondary disease, a significantly higher proportion had a venous cause in the LSPC group (69% vs. 41%), while immobility was given as the cause in more patients in the inpatient population (44% vs. 18%). There was a substantial minority of patients who were given other classifications in both groups, but more frequently in the inpatient study group (23% vs. 14%).

**Table 2.** Causes of Lymphedema in Participants with Chronic Edema

*Classification*	*LPCS*		*Hospital inpatient prevalence*		χ^2^*(df)*	p
	N	*%*	N	*%*		
Primary	94	13.0	2	1.1	21.02 (1)	<0.001
Secondary	629	87	175	98.9		
Total	723		177			
Cancer						
Present	55	8.7	1	0.6		<0.001^a^
Absent	574	91.3	174	99.4		
Total	629		175			
Venous						
Present	437	69.4	71	40.6	49.17 (1)	<0.001
Absent	192	30.5	104	59.4		
Total	629		175			
Immobility						
Present	111	17.7	77	44.0	53.07 (1)	<0.001
Absent	518	82.3	98	56.0		
Total	629		175			
Obesity						
Present	125	19.9	42	24.0	1.42 (1)	0.23
Absent	504	80.1	133	76.0		
Total	629		175			
Other						
Present	87	13.8	40	22.9	8.39 (1)	0.004
Absent	542	86.2	135	77.1		
Total	629		175			

^a^ Fisher's exact test.

### Location and duration of CO

Surprisingly, just over 6% of patients suffered from arm CO in both groups (Table 3). The difference between groups became apparent when examining the proportion of patients with leg and midline CO. More patients were found with midline CO in the LSPC group (30) compared with none in the inpatients.

**Table 3.** Location and Duration of Lymphedema in Participants with Chronic Edema

	*LPCS*		*Hospital inpatient prevalence*		χ^2^*(df)*	p
	N	*%*	N	*%*		
Arm (s)						
Present	46	6.4	11	6.2	0.005 (1)	0.94
Absent	677	93.6	166	93.8		
Total	723		177			
Leg (s)						
Present	683	94.5	176	99.4		0.002^a^
Absent	40	5.5	1	0.6		
Total	723		177			
Midline						
Present	30	4.2	0	0.0		0.002^a^
Absent	693	95.9	177	100.0		
Total	723		177			
Duration						
<6 months	74	10.2	40	22.6	29.10 (5)	<0.001
6 months to 1 year	97	13.4	13	7.3		
1–2 years	126	17.4	16	9.0		
2–5 years	152	21.0	33	18.6		
5–10 years	88	12.2	26	14.7		
10+ years	186	25.7	49	27.7		
Total	723		176			
Control of swelling						
Yes	469	66.7	35	19.9	126.2 (1)	<0.001
No	234	33.3	141	80.1		
Total	703		176			

^a^ Fisher's exact test.

In the inpatient study, we found that 23% of patients had CO for less than 6 months compared with 10% in the LSPC group. In those with a duration of 6–12 months and 1–2 years, the pattern was reversed with a higher percentage being present in the LSPC group. With longer duration of lymphedema, the groups had similar distribution. In both populations, over 26% had suffered with swelling for more than 10 years.

### Infection and hospitalization for CO

We found that 14% of patients in the hospital prevalence of CO have had an infection in the area of CO (Table 4) and almost 8% of the cases had this infection within the last year.

**Table 4.** Infections in Participants with Chronic Edema

	*LPCS*		*Hospital inpatient prevalence*		χ^2^*(df)*	p
	N	*%*	N	*%*		
Ever had cellulitis?						
Yes	221	30.6	25	14.1	19.35 (1)	<0.001
No	502	69.4	152	85.9		
Total	723		177			
Acute infection in the last year?						
Yes	143	19.8	14	7.9	13.90 (1)	<0.001
No	580	80.2	163	92.1		
Number						
1	84	58.7	9	64.3		
2	33	23.1	3	21.4		
3	11	7.7	1	7.1		
4	7	4.9	0	0		
5	5	3.5	1	7.1		
>5	3	2.1	0	0		
Total	723		177			
Hospitalized in the last year?						
Yes	73	10.1	11	6.2	2.53 (1)	0.11
No	650	89.9	166	93.8		
Number						
1	58	79.5	8	72.7		
2	10	13.7	1	9.1		
3	3	4.1	1	9.1		
>3	2	2.7	1	9.1		

In LSPCs, over 30% had a history of infection in the area of CO, and of the total group, 20% experienced an infection within the past year. The number of infections varied greatly, with the majority experiencing one or two infections.

Interestingly, while the number of infections was lower in the inpatient group, nevertheless, they were more likely to be admitted to hospital for the infection (11/14 vs. 73/143).

### Treatment and control of swelling

In the LSPC group, 83% were receiving some form of standard lymphedema treatment. This compares with just 27% in the hospital group (Table 5). These differences were noted for multilayer bandaging (43% vs. 7%), use of a compression garment (63% vs. 23%), and manual lymphatic drainage (5% vs. 0%).

**Table 5.** Treatments for Lymphedema and Control of Swelling

	*LPCS*		*Hospital inpatient prevalence*		χ^2^*(df)*	p
	N	*%*	N	*%*		
None	124	17.1	130	73.5	222.5 (1)	<0.001
At least one	599	82.9	47	26.6		
Total	723		177			
Multilayer bandaging						
Present	312	43.2	12	6.8	81.6 (1)	<0.001
Absent	411	56.9	165	93.2		
Total	723		177			
Compression garment						
Present	453	62.7	40	22.6	92.1 (1)	<0.001
Absent	270	37.4	137	77.4		
Total	723		177			
Massage						
Present	38	5.3	0	0.0		<0.001^a^
Absent	685	94.7	177	100		
Total	723		177			
Control of swelling						
Yes	469	66.7	35	19.9	126.2 (1)	<0.001
No	234	33.3	141	80.1		
Total	703		176			

^a^ Fisher's exact test.

Patients within the LSPC group were more likely to experience good subjective control of their swelling compared with inpatients (67% vs. 20%). This is perhaps not surprising given the paucity of treatments being offered to those patients within the hospital study.

## Random sample [inpatient population *N* = 116/177(65.5%)]

Data from the random sample from the hospital inpatient service indicated that 47/116 (41%) lived alone, with 56/116 (48%) owning their own property. Not surprisingly, given the age of the sample, 69% were retired with only 5% not working due to illness. However, 10% stated that the family income had been affected by having CO, irrespective of their working status. The majority were defined as having pitting edema with limb shape distortion evident in 32%.

### Feasibility of the methods

The study provided important insights into undertaking the study in diverse settings. Changes in education for the main study were made and this included developing a short video to assess pitting edema, performing a Stemmer test, and measuring the limb for shape distortion. Quality mechanisms for ensuring quality data were effective and integrated into the study standard operating procedures. The validation process reinforced the hypothesis that classification of lymphedema as primary and secondary lymphedema is difficult even when there is access to trained lymphologists and cannot be performed reliably in primary care settings.

## Discussion

The high prevalence of 39% of hospital inpatients suffering from CO has not been reported before and indicates that it is an important problem in hospital settings and is accompanied by a range of comorbidities. As admission was generally unrelated to CO, one might consider that these patients would be suffering from a mild condition, which does not require treatment. However, evidence from this study indicates that this is not the case. These patients were elderly with a range of comorbidities that might predispose them to a chronic and severe predisposition and the development of infection and other issues that characterize poor outcome.

Comparison between this group and a group who were receiving care from a variety of specialist lymphedema services and primary care services has identified not only similarities but also several differences. As an example, the inpatient group experienced fewer acute infections than those managed by the lymphedema services. However, when they did develop an infection, it more frequently led to hospitalization. It is difficult to know quite why this is so, but it is perhaps indicative of patients within the lymphedema services being managed more effectively without the need for hospital admissions.

Another key element of risk is that of poor mobility. From the data, it is clear that immobility was cited as a cause of CO more frequently in those admitted to hospital than those in dedicated lymphedema services. Again, it is hard to be precise on what is occurring, but this fits the profile of a more elderly immobile population receiving suboptimal care in the community. Clearly, this is speculation, which requires further investigation.

### Limitations

The study has some limitations that must be addressed. In particular, the participants recruited from community-based lymphedema services and primary care nursing services are unlikely to be representative of the total population receiving care within this service, leading to a selection bias. This led the LIMPRINT study team to develop specific methods to recruit for community studies to increase reliability and validity. These have been reported separately.^9^

## Conclusion

This initial validation study highlighted opportunities and challenges associated with undertaking LIMPRINT in a different health setting. It led to improvements in the study methodology and quality mechanisms. The hospital inpatient study showed the difficulty of gaining consent and accessing patients in busy hospital settings. Nevertheless, in those recruited, the prevalence of 39% is the highest yet recorded. Comparison of participant profiles between the inpatient and other lymphedema specialist services and primary care reveals a complex picture of comorbidities that have been examined further in other LIMPRINT studies.
